# Charting a Sustainable Course: *Phaeobacter* Inoculation as a Probiotic-Based Strategy for Common Octopus Aquaculture During Early Life Stages

**DOI:** 10.3390/microorganisms14051165

**Published:** 2026-05-21

**Authors:** Luana Granja, Jorge Carlos Santamaría, José Pintado, Camino Gestal, Gonzalo Del Olmo

**Affiliations:** 1 Integrated Marine Ecology (INMARE), Instituto de Investigacions Mariñas—CSIC, Rúa de Eduardo Cabello, 6, 36208 Vigo, Spain; luanagranjafz@gmail.com (L.G.); jsantamaria@iim.csic.es (J.C.S.); pintado@iim.csic.es (J.P.); 2 Marine Molecular Pathobiology (PatoBioMar), Instituto de Investigacions Mariñas—CSIC, Rúa de Eduardo Cabello, 6, 36208 Vigo, Spain

**Keywords:** *Octopus vulgaris*, probiotics, *Vibrio* infections, *Tenacibaculum maritimum*, host–microbe interactions, biological control

## Abstract

The aquaculture of *Octopus vulgaris* faces high larval mortality, mainly due to nutritional limitations and susceptibility to pathogens, particularly *Vibrio* spp. As vaccination is not feasible in cephalopods, host-associated probiotics represent a promising and sustainable alternative to improve survival and reduce infections. This study evaluated bacteria from the *Roseobacter* clade as probiotic candidates during octopus embryonic and paralarval stages. Characterization of egg-associated microbiota revealed the absence of cultivable bacteria within eggs and a significantly lower bacterial load on egg surfaces under maternal care, highlighting the regulatory role of female cleaning behavior. No bacteria with antagonism against *Vibrio lentus*, a common pathogen to octopus, were isolated from egg surfaces. Therefore, selected *Roseobacter* clade strains were screened in vitro against relevant aquaculture pathogens. *Phaeobacter* strains showed strong inhibitory activity against *Vibrio* spp., including *V. lentus*, while *Ruegeria* strains exhibited higher specificity against *Tenacibaculum maritimum*. Based on these results, *Phaeobacter* sp. 4UAC3 was selected for in vivo assays. This strain successfully colonized eggs, water, and paralarvae; however, its application reduced hatching success in eggs by 33%, likely due to surface-associated accumulation of the bacteria linked to the administration method. In contrast, probiotic treatment significantly improved survival at the paralarval stage. Although high variability was observed, probably due to stressful rearing conditions, more than 50% was observed in treated vs. 0% in non-treated cases at day 6. Overall, *Phaeobacter* sp. 4UAC3 emerges as a promising probiotic candidate to improve *O. vulgaris* paralarvae survival, potentially contributing to solving this bottleneck in a sustainable way.

## 1. Introduction

Aquaculture has expanded rapidly in recent decades due to the increasing global demand for seafood and the continuous growth of the human population. In 2022, aquaculture accounted for approximately 50% of global seafood production [1], highlighting its central role in ensuring food security. However, this expansion has also increased the need to develop more sustainable production systems that minimize environmental impact while improving animal health and welfare.

Within this context, cephalopod aquaculture has attracted increasing scientific and commercial interest. Among these species, the common octopus (*Octopus vulgaris*) is one of the most valuable cephalopods in European fisheries, particularly in Spain, where it represents an important cultural and economic resource [2]. Consequently, the development of sustainable aquaculture systems for *O. vulgaris* has become a priority in recent years. In addition to productivity, the welfare of cultured animals has also gained increasing attention, especially considering that cephalopods are the only invertebrates included in European legislation regulating the use of animals in research [3] due to their recognized capacity to experience pain and distress [4].

With the zootechnical obstacles to completing the common octopus life cycle in captivity now overcome [5,6], culture has been successfully implemented under experimental conditions in recent years, and the transition to industrial-scale production is in progress. Several studies have focused on optimizing their culture by improving diets, culture conditions, environmental factors, and behavior, as well as their general health [7,8,9,10], but standardized health and welfare practices still need further development.

One of the main bottlenecks in octopus farming has historically been the high mortality rate during the paralarval stage [5,11]. During this stage, which extends from hatching to approximately day 65 [12], the individuals lead a planktonic lifestyle: they swim actively and feed on live planktonic organisms [13]. From 30 days of age, the survival of paralarvae in culture declines rapidly, reaching values close to 20% by day 40 [14]. This is largely attributed to nutritional deficiencies, particularly the scarcity of lipids in the live prey used as feed in aquaculture [12].

In addition to zootechnical limitations, disease outbreaks represent another major constraint for successful octopus culture. Cephalopods rely exclusively on innate immunity and lack adaptive immune responses, which limits the development of preventive strategies such as vaccination [15]. Rearing systems, which typically involve high animal densities and increased organic matter, can promote bacterial proliferation and disease transmission. Among the most relevant bacterial pathogens affecting marine organisms are species of the genus *Vibrio*, which are responsible for vibriosis and skin lesions in several aquaculture species. In octopus, pathogens such as *Vibrio lentus*, *Vibrio alginolyticus*, *Vibrio parahaemolyticus*, and *Vibrio splendidus* have been associated with disease outbreaks and mortality [10,16].

In this context, probiotics have emerged as a promising strategy to improve health and survival in aquaculture systems while reducing the use of antimicrobial and disinfectant treatments and therefore increasing animal welfare. Probiotics are defined as live microorganisms that confer health benefits to the host when administered in adequate amounts [17]. In aquaculture, probiotics can modulate the immune system, inhibit pathogen growth, improve nutrient digestion, and enhance overall performance of cultured organisms [18]. In recent years, increasing attention has been given to host-associated probiotics, which are isolated from the natural microbiota of the cultured organism or from its environment [19]. These microorganisms are often better adapted to marine conditions and may establish more stable interactions with the host compared with terrestrial probiotic strains [20].

Among potential probiotic candidates in aquaculture, bacteria belonging to the *Roseobacter* clade (Alphaproteobacteria) have attracted particular interest [21]. This diverse and abundant group of marine bacteria, widely distributed in coastal environments [22,23,24,25], is known for its ability to colonize biotic and abiotic surfaces [26] and interact with marine eukaryotes [27,28,29,30]. In cephalopods, members of this clade have been detected in accessory nidamental glands and egg capsules of species such as *Loligo pealei* [31,32,33], *Sepia officinalis* [34], and *Euprymna scolopes* [35,36,37]. Where they are believed to contribute to the protection of developing embryos against microbial infections [37,38]. In octopuses, microbiome studies of *Octopus mimus* eggs have revealed a predominance of Alphaproteobacteria from the *Roseobacter* clade in healthy eggs [39,40], with genera such as *Leisingera* and *Sulfitobacter* also detected in paralarvae, as well as isolates of *Roseobacter* spp. in the digestive tract of adult specimens [41].

Several species within the *Roseobacter* clade, particularly *Phaeobacter* and *Ruegeria*, have demonstrated strong antagonistic activity against important aquaculture pathogens [42,43,44,45,46,47], and have been reported to not be detrimental for hosts when they are inoculated [47,48]. These bacteria are able to produce bioactive compounds such as tropodithietic acid (TDA), a secondary metabolite with broad antibacterial activity against several pathogenic bacteria, including species of the genus *Vibrio* [24]. The absence of induction of resistance in pathogens by TDA has been reported [24,49], likely due to its mode of action, namely, disruption of the proton motive force, an essential and highly conserved cellular process that is difficult to bypass through simple mutations [50]. In addition to its antimicrobial properties, TDA also functions as a signaling molecule involved in quorum sensing and regulation of bacterial behavior, including biofilm formation and surface colonization [51,52]. Given these characteristics, members of the *Roseobacter* clade represent promising candidates as probiotics for marine aquaculture, particularly for early developmental stages where disease susceptibility is high.

Although studies suggest that probiotics in octopus farming deserve further research [53], and some bacterial strains have been proposed as potential candidates [54,55], especially those from *Roseobacter* clade [40], to the authors’ knowledge, no studies have yet tested probiotics in octopus culture, including eggs and paralarvae rearing.

The present study aimed to evaluate bacteria belonging to the *Roseobacter* clade as potential probiotics to enhance survival and pathogen resistance during the early developmental stages of *O. vulgaris* culture. Specifically, the study first assessed the parental effect on egg-associated bacterial load and focuses on the isolation and identification of candidate probiotic strains from the *Roseobacter* clade associated with octopus’ eggs. As no antagonistic egg-associated bacteria were isolated, the in vitro inhibitory capacity of other *Roseobacter* bacterial strains against relevant aquaculture pathogens was examined to identify the most promising candidate. Finally, the study evaluated the in vivo efficacy of the selected strain through experimental trials in octopus egg culture and infection challenge assays using *O. vulgaris* paralarvae. Overall, probiotic-based approaches applied at early developmental stages of *O. vulgaris* hold considerable potential to enhance the health and welfare status of octopuses in aquarium and aquaculture systems.

## 2. Materials and Methods

### 2.1. Bacterial Strains and Culture Conditions

The probiotic strains evaluated in this study were *Phaeobacter* sp. 4UAC3 (CECT 31030), *Phaeobacter gallaeciensis* (CECT 7277), *Phaeobacter* sp. 27-4 (CECT 7251), *Ruegeria* sp. ALR6, and *Ruegeria* sp. LRC4. The pathogenic strains included *Vibrio anguillarum* (90-11-287, serotype O1), *Vibrio lentus*, *Vibrio splendidus* (LGP32), and *Tenacibaculum maritimum*. More information about the strains can be consulted in Appendix A.

All strains were stored at −80 °C in Marine Broth (MB, Difco™ 2219, Franklin Lakes, NJ, USA) supplemented with 15% glycerol, as previously described [56], except for *Vibrio splendidus* and *Vibrio lentus*, which were preserved at −80 °C in tryptic soy broth (TSB) with 15% glycerol.

Probiotic strains were inoculated into 4.5 mL of MB and incubated at 20 °C in darkness for 72 h (pre-culture). Subsequently, 450 μL of the bacterial suspension was transferred to fresh MB and incubated under the same conditions for 48 h (culture) [56]. For pathogenic strains, *Vibrio anguillarum* and *Vibrio splendidus* were inoculated into 4.5 mL of MB and incubated at 22 °C with agitation (110 rpm) in darkness for 48 h (pre-culture), followed by transfer of 45 μL of the suspension to fresh MB and incubation for 24 h (culture). *Vibrio lentus* was cultured under the same conditions, except that the final culture lasted 48 h. *Tenacibaculum maritimum* was pre-cultured for 72 h and subsequently for 48 h under the same conditions as the other pathogens.

### 2.2. Isolation of Bacteria from Octopus Vulgaris Eggs

In order avoid variability from clutches, egg clusters of *Octopus vulgaris* were obtained from the spawning (on May 2025) of a single wild female maintained in captivity at the Spanish Institute of Oceanography (IEO) and transported in proper containers to the Experimental Culture Facilities of IIM-CSIC, institution which is registered as “User and breeding center on animal experimentation” ES360570202001. Procedures for transportation and maintenance were carried out in accordance with the principles published in the European Directive (2010/63/EU) for the protection of experimental animals used for scientific purposes and were approved by the Spanish National Competent Authority ethics committee (Research Project ES360570202001/17–24/EDUCFORM 07/CGM01). After laying, egg strings were removed from the female and suspended in the water by tying the main cluster to a nylon wire support, maintaining a constant water flow that simulated the maternal siphoning behavior observed under natural conditions [57,58]. Eggs were maintained in darkness in 20 L tanks with continuous flow of seawater from the Ría de Vigo (Pontevedra, Spain) at approximately 15 °C, supplied through the IIM seawater distribution system. Prior to use, seawater was filtered through a sand filter followed by cartridge filters with pore sizes of 100 and 20 µm and subsequently sterilized using ultraviolet light.

To assess the presence of bacterial microbiota within *O. vulgaris* eggs, the internal contents of eight individual eggs (*n* = 8) were aseptically extracted using a stereomicroscope in sterile conditions. The internal content was transferred into a microcentrifuge tube containing 1 mL of sterile filtered seawater and homogenized by vortex for 2 min. Serial dilutions were subsequently prepared and plated onto Marine Agar (MA, Difco™ 2216, Franklin Lakes, NJ, USA), tryptic soy agar supplemented with 2% NaCl (TSA + 2% NaCl), and LB agar. Plates were incubated at 20 °C for 5 days.

To evaluate the probiotic potential of bacteria associated with the surface of *O. vulgaris* eggs, as well as the role of maternal care in shaping the composition and abundance of the egg-associated microbiota, egg clusters naturally ventilated by females were compared with clusters maintained under artificial aeration for approximately 10 days after removal from the maternal tank. All eggs originated from the same spawning event and the same female. Surface-associated bacteria were collected by swabbing the egg surface with sterile cotton swabs. Each swab was transferred into a sterile Eppendorf tube containing 1 mL of sterile seawater and vortex for 2 min. The resulting suspension was serially diluted and plated onto MA plates, which were incubated at 22 °C for 5 days. For each condition (“maternal siphoning” and “artificial aeration”), four independent replicates were analyzed, and four serial dilutions were plated. After incubation, colony-forming units (CFU) were counted.

Replica plating onto a *Vibrio lentus* lawn (10^3^ CFU·cm^−2^) was performed to evaluate the inhibitory capacity of the cultivable colonies. In parallel, colonies were selected based on pigmentation and morphology, particularly those showing characteristic brown pigmentation associated with members of the *Roseobacter* [59]. Selected colonies were purified and inoculated into cryotubes containing MB supplemented with 15% glycerol. Cultures were preserved through gradual freezing (4 °C, −20 °C, and finally −80 °C) for long-term storage. These isolates constituted the working stock for subsequent assays evaluating potential probiotic activity by Well Diffusion Agar Assays (WDAAs). However, no inhibitory activity against any pathogen was detected in either assay.

### 2.3. In Vitro Assays: Well Diffusion Agar Assay

As no antagonistic bacteria were successfully isolated from *O. vulgaris* eggs under the culture conditions applied, the inhibition assays were subsequently performed using previously characterized probiotic strains belonging to the *Roseobacter* clade (Appendix A). The antimicrobial activity of these strains against aquaculture-relevant pathogenic bacteria was evaluated using the WDAA, a method widely applied in aquaculture research involving *Phaeobacter* spp. [60,61].

The MA was inoculated with the pathogenic strains (≈10^5^ CFU·mL^−1^) at 44 °C. 45 mL was poured per plate and six wells with a sterile punch was created. Twelve replicate plates were prepared for each of the four pathogenic strains. Then, 30 µL of each probiotic culture (sterile MB was used as a negative control) was added to the plates, which were then incubated at 20 °C in the dark for 72 h. The inhibition halos were then measured using Fiji (ImageJ, version 2.17.0) [62] on images obtained with a stereomicroscope.

### 2.4. In Vivo Assays

The in vivo trials were designed to evaluate the effect of *Phaeobacter* sp. 4UAC3 across two consecutive developmental stages of *O. vulgaris*: the embryonic stage and the paralarval stage. In the first phase, egg clusters were exposed to *Phaeobacter* sp. 4UAC3 suspensions through repeated immersion baths, as described in Section 2.4.1. In the second phase, paralarvae hatched from both treated and untreated eggs were used as subjects in an infection challenge assay as described in Section 2.4.2. Given that persistent colonization of paralarvae by *Phaeobacter* sp. 4UAC3 following hatching could not be assumed, a direct inoculation of the probiotic was performed in the corresponding treatment groups before pathogen exposure.

#### 2.4.1. Probiotic Immersion Treatments Applied to Octopus’ Eggs

Prior to the initiation of the experimental treatments, total number of eggs per cluster (experimental unit) was estimated. One side of each cluster was photographed in sections, and the images were analyzed using Fiji (ImageJ). The total number of eggs per cluster was estimated by doubling the number counted. Treatments began when the first signs of ocular development (Stage VIII) were observed, according to the developmental stages described by Deryckere (2020) [58].

**Figure 1:**
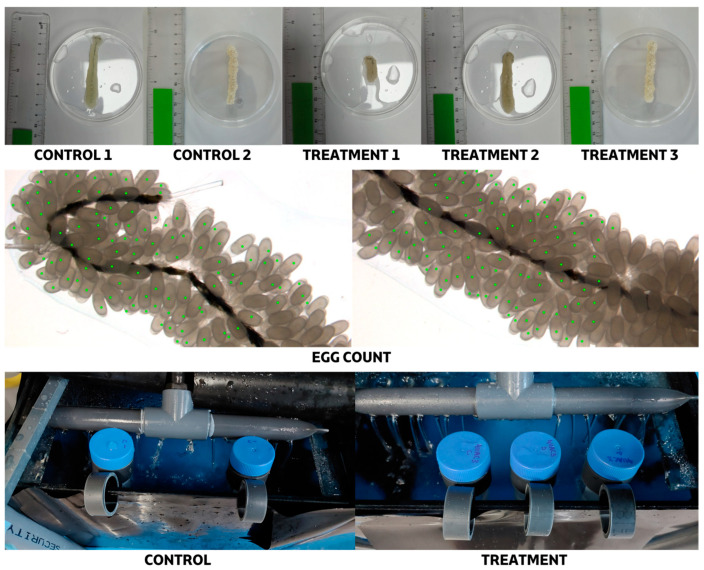
Experimental procedure for Falcon tube baths. The figure shows the *Octopus vulgaris* egg clusters used in each experimental condition, the image-based quantification (eggs with green dots), and the artificial siphoning culture system, including the supports employed during the bath treatments.

The experimental design included two conditions. In the control condition, eggs were exposed only to sterile artificial seawater (ASW) to verify that handling procedures did not affect egg survival. This group consisted of two clusters (Control 1 and Control 2) maintained together in the same tank. In the treatment condition, eggs were exposed to bacterial suspensions of *Phaeobacter* sp. 4UAC3, based on the results from in vitro assays. Three clusters (Treatment 1, Treatment 2, and Treatment 3) were included in this group. Swab samples were collected only from cluster Treatment 3 to confirm bacterial colonization on the egg surface. As swabbing could potentially affect hatching, only this smaller cluster (Figure 1) was sampled, while clusters 1 and 2 were left undisturbed.

The *Phaeobacter* sp. 4UAC3 culture was prepared following the protocol described previously. Once cultures reached the stationary phase (≈10^8^ CFU·mL^−1^), 5 mL of culture per replicate was centrifuged at 12,000 rpm for 10 min. The resulting pellet was resuspended in 50 mL of sterile ASW to obtain a final concentration of ≈10^7^ CFU·mL^−1^, which was verified by plating in MA.

For the immersion procedure, bacterial suspensions and control solutions (sterile ASW) were placed in 50 mL Falcon tubes (Figure 1) and kept in the egg tank for 20 min prior to the assay to equilibrate to the same temperature. Egg clusters were then immersed in the respective suspensions for 1 h. A total of ten immersion treatments were conducted over a five-week period, from 16 May to 11 July 2025. To monitor cluster growth and morphological development, egg clusters were weighed and photographed prior to each immersion treatment.

Additional swab samples were collected after the initiation of immersion treatments (2 and 7 days after the first treatment) to quantify bacterial load on the egg surface and assess colonization by *Phaeobacter* sp. 4UAC3. In media with high ferric iron (Fe^3+^) availability, such as marine broth supplemented with ferric citrate, TDA forms an insoluble complex [Fe^3+^(TDA)_2_]_x_ that appears as a brown pigment [42], facilitating the identification of TDA-producing colonies during culture.

Once all *O. vulgaris* paralarvae had hatched, hatching success was determined by manually counting the number of unhatched eggs under a stereomicroscope. These eggs were classified into three categories according to their developmental stage: early stages (Stages I–VIII), with no observable embryonic development; intermediate stages (Stages VIII–XIV), with visible red eye spots; and advanced stages (Stages XV–XX), where embryos displayed chromatophores or other clearly visible internal structures [58]. This classification allowed evaluation not only of the overall hatching rate but also of the developmental status of unhatched eggs.

#### 2.4.2. Infection Assay of Octopus paralarvae Culture

To further investigate the protective effect of *Phaeobacter* sp. 4UAC3, an in vivo infection trial with *V. lentus* was carried out using *O. vulgaris* paralarvae.

**Figure 2:**
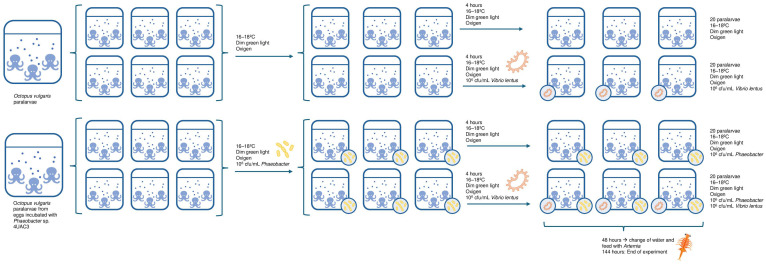
Schematic diagram of the experimental design for the *O. vulgaris* paralarvae infection assay. Figure created using Microsoft PowerPoint and ChatGPT (GPT-5.5, OpenAI, 2026).

Paralarvae were obtained from a spawning event on 13 May 2025, with hatching beginning on 11 July 2025. Individuals used in the experiment were approximately 5–7 days post-hatching. Paralarvae used in the control and infection treatments originated from untreated eggs, whereas those used in probiotic-related treatments originated from eggs previously immersed with *Phaeobacter* sp. 4UAC3 suspensions. Four experimental conditions were established (Figure 2): (i) control (paralarvae only), (ii) *V. lentus* infection, (iii) probiotic control (inoculation with *Phaeobacter* sp. 4UAC3), and (iv) probiotic + *V. lentus*. Each condition was performed in triplicate, resulting in a total of 12 aquaria containing 20 paralarvae per aquarium based on the paralarvae available. The experiment was conducted in an infection room maintained at 18 °C under dim green light, using 1 L aquaria previously disinfected with bleach and thoroughly rinsed. Each aquarium contained 500 mL of autoclaved seawater (121 °C, 20 min) with gentle aeration.

Six aquaria were inoculated with *Phaeobacter* sp. 4UAC3 (T−4 h) to reach a final concentration in the tank of 10^6^ CFU mL^−1^. After 4 h of exposure (T0 h), infection was performed by adding *Vibrio* culture to achieve a final concentration of 10^6^ CFU mL^−1^. Sampling times were established at T−4 h (before *Phaeobacter* inoculation), T0 h (after *Phaeobacter* and before *Vibrio* infection), T24 h, T48 h, and day 6 (end of the experiment).

Prior to inoculation (T–4 h), no differences were detected between groups in the concentration of total cultivable bacteria, which could indicate that the initial conditions were microbiologically homogeneous. The absence of *Phaeobacter*, even in paralarvae from previously treated eggs, could be due to the initial colonization not persisting after hatching, the probiotic failing to reach the interior of the egg, or it being present at undetectable levels.

Initially, paralarvae were not fed to avoid alterations associated with the microbiota of *Artemia* and the accumulation of organic debris. After 48 h, part of the water was replaced with filtered seawater from the Ría de Vigo, as previously described. Considering the limited yolk reserves of the paralarvae and their reduced capacity to survive prolonged periods without feeding, enriched *Artemia* were subsequently provided after 48 h, according to their nutritional requirements [63].

Paralarvae survival was monitored daily throughout the experiment, and dead individuals were removed.

For microbiological analyses, samples were collected in triplicate by randomly selecting one larva per aquarium at each sampling time. Larvae were euthanized following Directive 2010/63/EU using an initial anesthetic solution (1.5% MgCl_2_ and 1% ethanol in seawater for 5 min), followed by an overdose of the anesthetic (3%) [3]. Each dead larva was homogenized in 300 µL of sterile seawater using a micropestle, plated on MA, and incubated at 20 °C in darkness for 72 h.

### 2.5. Statistical Analysis

For the bacterial load analyses (log_10_ CFU per egg in each cluster), normality of data distributions for each group (Artificial Siphoning and Maternal Siphoning) was assessed using the Shapiro–Wilk test, and homogeneity of variances was evaluated using Levene’s test. As both assumptions were satisfied, differences between groups were analyzed using a Student’s *t*-test.

For the in vitro inhibition assays, data were structured according to the pathogen tested and the probiotic strains evaluated to identify those with the highest inhibitory capacity and to detect differences in antimicrobial activity. Additionally, to facilitate comparisons among treatments, inhibition halo areas (mm^2^) were normalized by the concentration of probiotic applied, expressing inhibitory capacity per unit of probiotic concentration. The objective of the analysis was to compare the magnitude of inhibition among strains that showed antagonistic activity; therefore, cases with no detectable inhibition were excluded from statistical analyses. As the data did not meet the assumptions of normality and homoscedasticity, non-parametric methods were applied. Differences among groups were assessed using the Kruskal–Wallis test, followed by Dunn’s post hoc test with Bonferroni correction for pairwise comparisons when significant effects were detected.

For the in vivo challenge experiment, regarding the egg trial, no statistical analyses could be carried out as there were not enough replicates; only the hatching rates and the percentages of embryonic stages in the unhatched eggs were calculated. As for the weight of the eggs, a linear regression was applied for each cluster over time solely to visually evaluate the course of egg growth. *O. vulgaris* eggs show little change in weight during early development, followed by an increase at mid-to-late stages due to water uptake [64]. Since measurements in this study began at an advanced developmental stage, only a limited part of the growth trajectory was observed, where the relationship between egg weight and time can be approximated as linear.

Regarding the paralarvae assay, survival of *O. vulgaris* paralarvae was analyzed using time-to-event models, accounting for both mortality events and censored observations (larvae collected alive for microbiological analyses). For each individual, time to event (death or censoring) was recorded, and event status was coded as 1 (death) or 0 (censored). Survival curves for each treatment were estimated using the Kaplan–Meier method, which has been widely applied in aquaculture survival studies [65,66,67]. Survival analyses were performed using the survival package in R, and curves were visualized with 95% confidence intervals using the Survminer R package [68]. Differences among treatments were evaluated using the log-rank (Mantel–Cox) test. Additionally, pairwise comparisons between treatments were conducted using pairwise log-rank tests, applying the Benjamini–Hochberg correction to control the false discovery rate.

All statistical analyses were performed in R software (version 4.3.1), and statistical significance was set at *p*-value (*p*) < 0.05.

## 3. Results

### 3.1. Effect of Parental Care on the Cultivable Microbiota of Octopus Eggs

**Figure 3:**
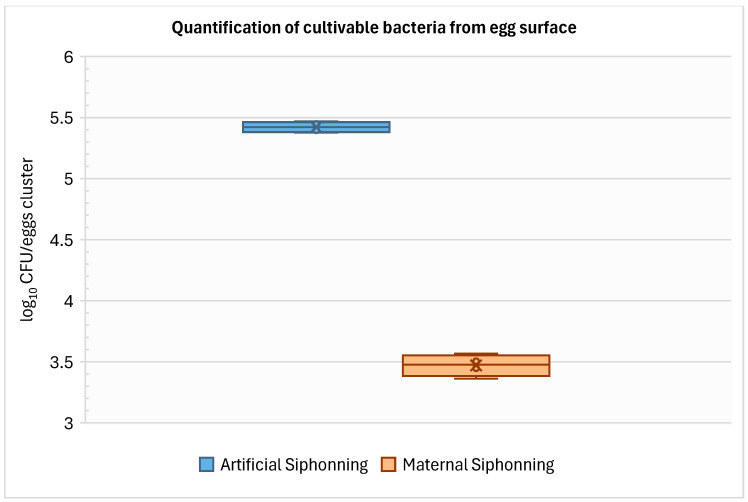
Quantification of cultivable bacteria from egg surface with and without progenitor care. Boxplots show the median (central line), the mean (cross sign), the interquartile range (box, 25th–75th percentile) and whiskers representing the minimum and maximum values.

Quantifications of cultivable bacteria on the egg surface were significantly different between eggs subjected to artificial siphoning and those receiving parental siphoning (Student’s *t*-test, *t* = 39.90, *p* = 1.66 × 10^−8^). Eggs subjected to artificial siphoning showed a mean abundance of 5.42 ± 0.04 log_10_ CFUs·cluster^−1^ (mean ± SD, *n* = 4), whereas eggs receiving parental care exhibited significantly lower values, averaging 3.47 ± 0.09 log_10_ CFUs·cluster^−1^ (mean ± SD, *n* = 4) (Figure 3).

The samples taken from inside the eggs had no cultivable bacteria in MA, LBA or TSA, while a total of 22 bacteria were isolated from the samples taken from the surface of the eggs (13 from samples without parental care and 9 with parental care), most of which appeared brown in MA culture (Supplementary Table S1), potentially Roseobacter clade bacteria producing TDA. However, the WDAA with *V. lentus* from the cultures of these isolates did not generate inhibition halos in any case. Also, no inhibition halos were observed in the replica plating tests on *V. lentus* lawn from the first plating of the bacteria from the egg surface for both conditions.

### 3.2. Inhibitory Capacity of Selected Roseobacter Clade Bacteria

**Figure 4:**
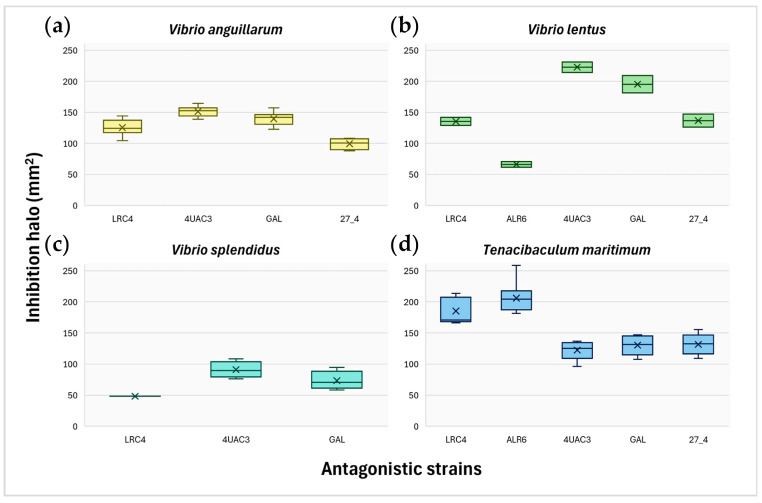
Absolute quantification of inhibition halos (mm^2^) produced by antagonistic strains (LRC4: *Ruegeria* sp. LRC4, ALR6: *Ruegeria* sp. ALR6, 4UAC3: *Phaeobacter* 4UAC3, GAL: *Phaeobacter gallaeciensis*, 27_4: *Phaeobacter piscinae* 27/4) against aquaculture pathogens: (**a**) *Vibrio anguillarum*, (**b**) *Vibrio lentus*, (**c**) *Vibrio splendidu*, and (**d**) *Tenacibaculum maritimum*.Boxplots show the median (central line), the mean (cross sign), the interquartile range (box, 25th–75th percentile) and whiskers representing the minimum and maximum values.

The probiotic strains tested (*Phaeobacter* sp. 4UAC3 CECT 31030, *P. gallaeciensis* CECT 7277, *Phaeobacter* sp. 27-4 CECT 7251, *Ruegeria* sp. ALR6, and *Ruegeria* sp. LRC4) showed variable inhibitory activity against the tested fish pathogens (Figure 4, Supplementary Figure S1). Most strains inhibited the growth of *V. anguillarum*, except *Ruegeria* ALR6. The largest inhibition halos were produced by *Phaeobacter* 4UAC3, followed by *P. gallaeciensis*. However, when inhibition was normalized to probiotic concentration (mm·CFU^−1^·mL), *P. gallaeciensis* showed the highest inhibitory potential, followed by *Phaeobacter* 4UAC3 and *Ruegeria* RLC4 (Supplementary Figure S2). Significant differences among strains were detected (Kruskal–Wallis, W = 25.81, *p* = 1.04 × 10^−5^), with Dunn’s test showing significant pairwise differences (*p* < 0.05), except between *Phaeobacter* 27/4 and *Ruegeria* LRC4, which exhibited the lowest inhibitory performance (Figure 4a).

All strains inhibited *V. lentus*. *Phaeobacter* 4UAC3 produced the largest inhibition halos, whereas *P. gallaeciensis* showed the highest inhibition relative to probiotic concentration (Figure 4b; Supplementary Figure S2). However, differences among strains were not statistically significant (Kruskal–Wallis, W = 8.29, *p* = 0.08).

Inhibition was observed against *V. splendidus* for all strains except *Ruegeria* ALR6. *Phaeobacter* sp. 4UAC3 generated the largest halos, while *P. gallaeciensis* again showed the highest inhibition relative to concentration (Figure 4c; Supplementary Figure S2). No significant differences among strains were detected (Kruskal–Wallis, W = 4.07, *p* = 0.13) among the three active probiotics.

All strains inhibited *Tenacibaculum maritimum*, albeit with marked differences in halo size (Kruskal–Wallis, W = 32.33, *p* = 1.64 × 10^−6^). The largest halos were produced by *Ruegeria* sp. ALR6 and *Ruegeria* sp. RLC4, with no significant differences between them but significant differences compared with the remaining probiotics according to Dunn’s test (Figure 4d; Supplementary Table S2). When normalized by probiotic concentration, *P. gallaeciensis* showed the highest relative inhibitory halos, followed by *Ruegeria* sp. ALR6 and *Ruegeria* sp. LRC4 (Supplementary Figure S2).

Based on these results, *Phaeobacter* sp. 4UAC3 was selected for the in vivo trials. This strain produced the largest inhibition halos in absolute terms against the majority of the pathogens tested and showed inhibitory activity against all four pathogenic species evaluated. Furthermore, previous experience with this strain in infection challenge assays [48] supported its selection for subsequent in vivo experimentation.

### 3.3. Effect of Phaeobacter sp. 4UAC3 Baths in Octopus’ Eggs

**Figure 5:**
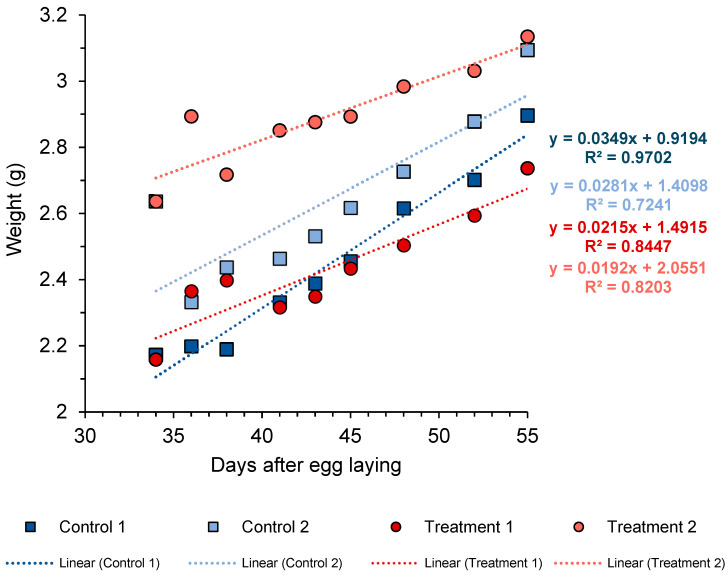
Evolution of octopus egg cluster weight during the *Phaeobacter* sp. 4UAC3 bath exposure assay. Blue squares: control groups; red circles: treatment (*Phaeobacter* baths).

**Figure 6:**
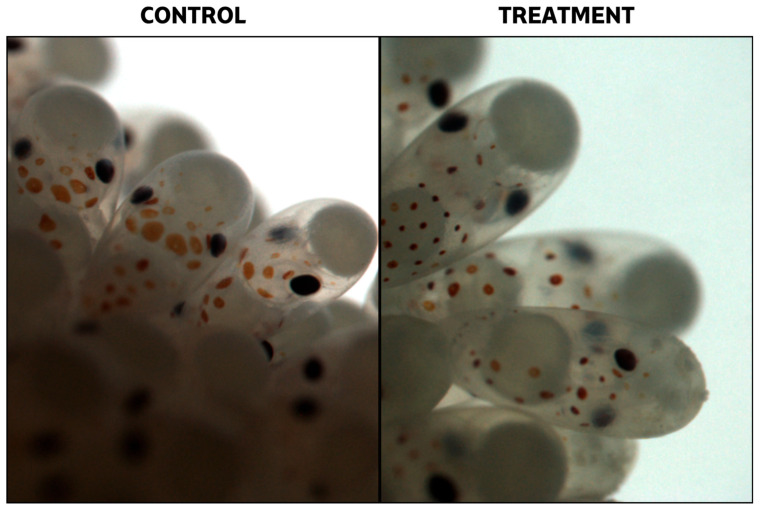
Comparison of *Octopus vulgaris* eggs under control conditions and after probiotic bath treatment. Black arrows highlight the presence of surface-associated material on treated eggs, absent in control samples.

Changes in egg cluster weight over time showed two distinct trends between control and treatment groups (Figure 5). Control clutches exhibited a higher growth rate, with slopes of 0.0349 (Control A) and 0.0281 (Control B), whereas treated groups showed lower values, with slopes of 0.0215 (Treatment 1) and 0.0192 (Treatment 2). Despite these differences in weight increase, embryonic development progressed similarly across treatments. No visual differences were observed among groups, except for a developmental delay of two weeks in the Treatment 2 group and, more markedly, in Control 1 (Supplementary Figure S3). In the final weeks of the experiment, however, eggs from the treated groups showed the presence of superficial layers of debris or organic matter on some egg surfaces (Figure 6).

The estimated initial number of eggs per cluster ranged from 942 to 1014 eggs, with the exception of group Treatment C, which contained a substantially lower number (360 eggs) (Table 1). Hatching success was consistent within both control and treatment groups but differed between them. Control groups showed high hatching rates (96–97%), whereas treated groups exhibited reduced values, with hatching rates between 63% and 64% (Table 2). Analysis of unhatched eggs according to the embryonic stage at which development ceased revealed contrasting patterns between treatments. In the control groups, most mortality occurred during early developmental stages (Stages I–VIII, approximately 58%), whereas in the treated groups, the majority of unhatched eggs had progressed to advanced developmental stages before arrest (Stages XV–XX, approximately 71%) (Table 2).

Microbiological analyses confirmed the presence of *Phaeobacter* sp. 4UAC3 in treated clusters. Swab samples were only taken from cluster from Treatment 3 and yielded 5.62 log_10_ CFU 48 h after the first treatment bath, increasing to 5.87 log_10_ CFU after seven days. Water samples from the Treatment 3 tank contained 2.27 log_10_ CFU·mL^−1^ of *Phaeobacter* sp. 4UAC3, together with similar concentrations of other cultivable microorganisms. *Phaeobacter* sp. 4UAC3 was not detected in the control tank, where the abundance of other cultivable microorganisms was approximately one order of magnitude lower.

### 3.4. Protective Effect of Phaeobacter sp. 4UAC3 on Octopus vulgaris Paralarval Survival During Pathogen Challenge Assays

#### 3.4.1. Paralarvae Survival

No relevant changes in larval survival were observed during the first 24 h of the infection challenge. During this period, the control, *V. lentus*, and *Phaeobacter*–*V. lentus* groups maintained 100% survival, whereas the remaining treatment (*Phaeobacter* alone) showed only a slight decrease (–3.33 ± 5.77%). Statistical analysis using the Kruskal–Wallis test confirmed the absence of significant differences among treatments (W = 3.00 *p* = 0.392).

At 48 h post-infection, differentiated trends began to emerge, accompanied by high variability among replicates. The highest mean survival percentages were recorded in the probiotic-inoculated groups, *Phaeobacter* (96.8 ± 6.79%) and *Phaeobacter*–*V. lentus* (92.16 ± 6.79%). In contrast, a marked reduction was observed in the control (62.45 ± 38.49%) and *V. lentus* (59.52 ± 52.65%) groups. However, these differences were not statistically significant (Kruskal–Wallis, W = 2.74, *p* = 0.434).

At 72 h, the same pattern persisted. The treatments inoculated with *Phaeobacter* displayed the highest mean survival values (*Phaeobacter*: 85.71 ± 14.29%; *Phaeobacter*–*V. lentus*: 83.33 ± 17.98%). Conversely, the control and *V. lentus* groups showed pronounced declines, with mean survival values of 19.05 ± 27.04% and 21.21 ± 29.22%, respectively, and complete mortality in at least one replicate. At this time point, the Kruskal–Wallis test identified significative differences (W = 8.52, *p* = 0.036), although Dunn’s post hoc test did not detect significant pairwise differences (Supplementary Table S3).

**Figure 7:**
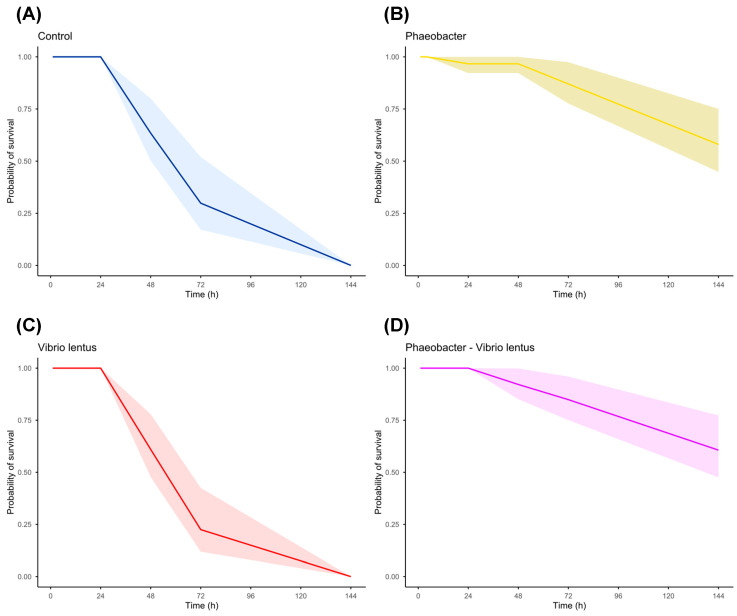
Survival curves with 95% confidence intervals (colored shadow) estimated using the Kaplan–Meier method for (**A**) control, (**B**) *Phaeobacter*, (**C**) *Vibrio lentus*, and (**D**) *Phaeobacter*–*Vibrio lentus* treatments. The graph was generated in RStudio (version 4.3.1).

After 6 days, the most pronounced divergences were observed. Both the control and *V. lentus* groups exhibited 100% mortality across all replicates (Figure 7). In contrast, treatments containing *Phaeobacter* retained substantially higher survival percentages: *Phaeobacter*–*V. lentus* (59.52 ± 39.34%) and *Phaeobacter* (57.14 ± 31.13%). The Kruskal–Wallis test indicated significant overall differences among treatments (W = 9.60, *p* = 0.022); however, Dunn’s test did not identify pairwise comparisons with *p* > 0.05 (Supplementary Table S4), probably due to the high variabilities observed.

Based on these results, survival curves were generated using the non-parametric Kaplan–Meier estimator (Figure 7). The curves again showed that by the end of the experiment, the highest survival rates corresponded to treatments containing the probiotic. Differences between survival curves were subsequently evaluated using the Log-Rank (Mantel–Cox) test (Supplementary Table S4). Survival analysis revealed significant differences between most treatments, with the most pronounced contrasts observed between Control and *Phaeobacter*, and between *V. lentus* and *Phaeobacter*–*V. lentus*.

#### 3.4.2. Bacterial Dynamics

**Figure 8:**
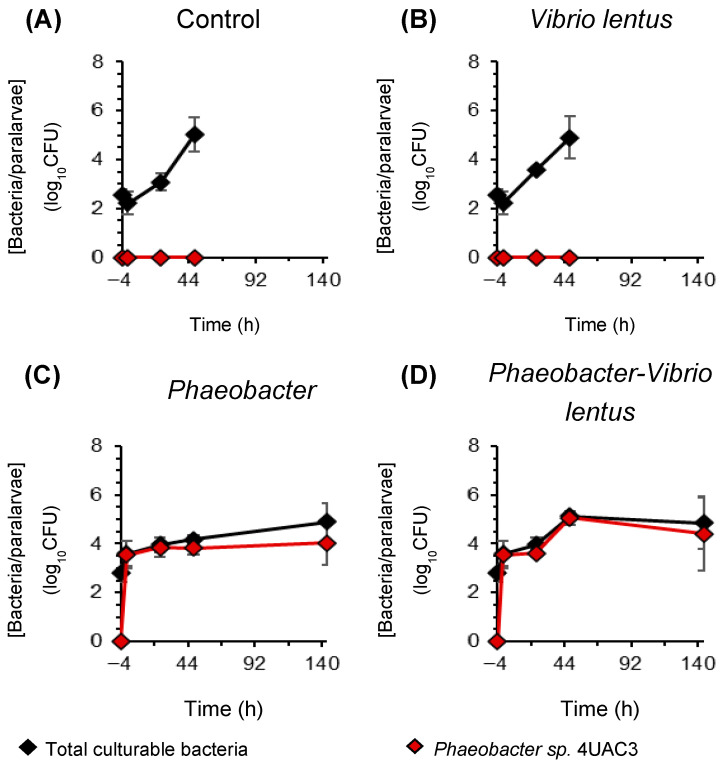
Microbial dynamics during the infection assay in octopus paralarvae in different conditions (**A**) Control, (**B**) *Vibrio lentus*, (**C**) *Phaeobacter*, (**D**) *Phaeobacter*-*Vibrio lentus*. Colony-forming unit (CFU) counts on Marine Agar are shown for total culturable bacteria (black diamonds) and *Phaeobacter* sp. 4UAC3 (red diamonds).

Total culturable bacterial concentrations in octopus paralarvae increased progressively during the infection challenge, with observed differences among treatments after probiotic supplementation (Figure 8). Prior to probiotic addition (–4 h), bacterial concentrations were low and similar across groups, reaching 2.57 ± 0.23 log_10_CFU per paralarva in individuals without previous exposure to *Phaeobacter* and 2.57 ± 0.23 log_10_CFU per paralarva in larvae originating from eggs previously treated with the probiotic. No *Phaeobacter* colonies were observed in this initial paralarvae and no significant differences among groups were detected (Kruskal–Wallis, W = 1.73, *p* = 0.631).

Four hours later (0 h), before pathogen infection, total bacterial concentrations remained low in non-inoculated groups (2.22 ± 0.45 log_10_CFU per paralarva), whereas probiotic-supplemented groups showed a markedly higher bacterial load, reaching 3.59 ± 0.51 log_10_CFU per paralarva. Differences among treatments were significant (Kruskal–Wallis, W = 8.49, *p* = 0.037), although pairwise comparisons were not significant (Dunn’s test *p* ≥0.05, Supplementary Table S5).

During the first 24 h post-infection, total bacterial concentrations increased in all treatments. The control group showed the lowest values (3.08 ± 0.36 log_10_CFU per paralarva), followed by the *V. lentus* infection group (3.56 ± 0.09 log_10_CFU per paralarva). Higher concentrations were observed in probiotic-supplemented treatments, reaching 3.96 ± 0.30 log_10_CFU per paralarva in the probiotic-only group and 3.96 ± 0.30 log_10_CFU per paralarva in the *Phaeobacter*–*V. lentus* group. Differences among treatments were significant (Kruskal–Wallis, W = 8.23, *p* = 0.041), and pairwise comparisons only detected significant differences between control and *Phaeobacter*–*V. lentus* (Dunn’s test *p* = 0.028, Supplementary Table S5).

After 48 h, total bacterial concentrations further increased across all treatments, reaching values between 4.17 ± 0.19 and 5.13 ± 0.09 log_10_CFU per paralarva. The lowest concentration was recorded in the probiotic-only inoculation group whereas the highest value was observed in *Phaeobacter*–*V. lentus* group (Figure 8). However, these differences were not statistically significant (Kruskal–Wallis, W = 3.68, *p* = 0.298).

Six days post-infection, after renewal with non-sterile estuarine seawater and the introduction of *Artemia* as feed, microbiological analyses were performed only in probiotic-treated groups due to the high mortality observed in non-treated situations. Mean total bacterial concentrations ranged from 4.91 ± 0.74 log_10_CFU per paralarva in *Phaeobacter*-only samples to 4.84 ± 1.06 log_10_CFU per paralarva in *Phaeobacter*-*V. lentus* samples, with no significant differences among treatments (Kruskal–Wallis, W = 1.19, *p* = 0.275).

Regarding the probiotic strain, *Phaeobacter* sp. 4UAC3 was not detected before probiotic supplementation, including in paralarvae originating from previously treated eggs. At 0 h, *Phaeobacter* sp. 4UAC3 was detected only in supplemented groups, reaching a mean abundance of 3.55 ± 0.57 log_10_CFU per paralarva and representing the majority of the culturable bacterial community. During the following 24 h, *Phaeobacter* sp. 4UAC3 remained detectable in all supplemented treatments, with mean abundances of 3.85 ± 0.41 log_10_CFU per paralarva in the probiotic-only group and 3.62 ± 0.14 log_10_CFU per paralarva in the *Phaeobacter*–*V. lentus* treatment, without significant differences among treatments (Kruskal–Wallis, W = 0.43, *p* = 0.513).

After 48 h, *Phaeobacter* abundance increased in infection treatments, reaching 5.07 ± 0.28 log_10_CFU per paralarva in the *Phaeobacter*–*V. lentus* group, whereas lower values were observed in the probiotic-only treatment (3.82 ± 0.27 log_10_CFU per paralarva). Differences among treatments were significant according to the Kruskal–Wallis test (W = 3.97, *p* = 0.046). By day 6, *Phaeobacter* sp. 4UAC3 concentrations decreased in the infection treatment (4.40 ± 1.53 log_10_CFU per paralarva), while remaining relatively stable in the probiotic-only group (4.02 ± 0.91 log_10_CFU per paralarva), with no significant differences among groups (Kruskal–Wallis, W = 0.43, *p* = 0.513).

## 4. Discussion

This study investigated the potential application of probiotic bacteria to improve survival and heath in the early development of the common octopus, from the embryonic stage to the paralarvae phase.

### 4.1. Microbial Load of Octopus Eggs and Maternal Influence

Previous studies have reported the presence of potentially beneficial bacteria, particularly from the *Roseobacter* clade, on the surfaces of eggs of various cephalopod species [31,34,35,40]. However, our results in *O. vulgaris* showed that although the egg surfaces contain cultivable bacterial communities, none of the isolates obtained exhibited antagonistic activity against *V. lentus*, a significant pathogen for cephalopods [69]. This absence of inhibitory activity could be explained by several factors, such as the impossibility of isolating the specific strains responsible for this activity, the ability of these bacteria to antagonize pathogens other than those used in the test, or the limitations associated with the cultivation of certain environmental bacteria that do not always grow in conventional culture media [70]. Analysis of the microbial community through 16S sequencing could provide a more complete picture of the bacterial diversity present on the egg surface. The interior of the eggs contained no cultivable bacteria, suggesting that it is a sterile environment. This finding is consistent with previous observations in other cephalopods, which report that no bacteria associated with either the embryo or the yolk [33].

A particularly relevant result was the lower bacterial load between eggs with maternal siphoning compared to eggs incubated under artificial siphoning. This result is consistent with the reproductive behavior of octopuses, as females invest considerable effort in caring for their egg laying [10]. Such care not only protects the eggs from predators but, by constant siphoning, also limits the proliferation of potentially harmful microorganisms, including bacteria and fungi associated with fouling or infection. Females continuously ventilate the eggs with seawater and mechanically clean their surface [58]. In addition, they remove eggs that show signs of infection, thanks to sensitive chemoreceptors in their arms, preventing the spread of pathogens to the rest of the eggs [39]. Along with these physical actions, a chemical defense is also involved, since maternal mucus contains defense-related proteins and antimicrobial peptides [71].

In some cephalopods, microbial protection of the eggs is also mediated by the transfer of symbiotic bacteria from the mother through the accessory nidamental glands (ANG; [31,34,35]). These glands host bacterial communities capable of producing antimicrobial compounds [36,37] that end in the gelatinous capsule that surrounds the eggs. However, octopuses lack these glands [10], and their eggs do not develop the thick gelatinous capsule typical of many other cephalopods. Instead, they are protected only by a thin, transparent chorionic layer [58]. As a result, the microbial colonization of octopus eggs likely depends largely on the surrounding environment rather than on maternal bacterial transfer [72]. This characteristic could explain the absence of probiotic bacteria detected on the surface of the eggs analyzed in this study, both in eggs with maternal care and in those incubated artificially. It is possible that the experimental environment, with treated seawater, did not contain a wide range of bacterial diversity to include specific pathogen-antagonistic bacteria that could colonize and protect the eggs, or that these bacteria are absent or present only in low abundance in the natural microbiome of *O. vulgaris* females. In fact, a recent study on the skin microbiome of this species identified the order Rhodobacterales as a sex-specific bacteria, being significantly more abundant on the skin of males than females [73], which could limit its maternal transfer to the eggs. In line with this hypothesis, although bacteria from the *Roseobacter* clade have been reported in the eggs of other species such as *Octopus minimus* [39], there is currently no clear evidence of their presence in the eggs of *O. vulgaris*.

### 4.2. Selection of Roseobacter Clade Strains as Probiotic Candidates

The absence of antagonistic bacteria on the surface of the eggs led us to explore other probiotic candidates. For this purpose, several strains belonging to the *Roseobacter* clade, previously isolated from marine environments (Appendix A), were evaluated for their potential to protect octopus’ eggs and paralarvae.

As a first step, the antagonistic activity of these strains was tested in vitro against several relevant aquaculture pathogens (Appendix A) in order to identify the most suitable candidate for further experiments. Based on these results, the strain *Phaeobacter* sp. 4UAC3 was selected for the in vivo trials. This choice was supported not only by the size of its inhibition halos but also by its favorable growth profile and by previous experience with this strain in the laboratory [30,48,61]. Although *P. gallaeciensis* showed higher relative inhibitory efficacy, its slower growth limits its practical application under culture conditions. In aquaculture systems, the ability to produce large amounts of a probiotic in the short term is an important factor in its final implementation. The differences between *Phaeobacter* sp. 4UAC3 and *P. gallaeciensis* could be related to variations in the production of TDA. It is possible that *P. gallaeciensis*, despite its slower growth, produces higher amounts of TDA, which would explain the formation of inhibition halos comparable to those produced by *Phaeobacter* sp. 4UAC3. In contrast, *Phaeobacter* sp. 4UAC3 may allocate more metabolic resources to cell growth, resulting in lower relative TDA production [47]. Furthermore, the differences observed in the in vitro trials may reflect the specific action of different probiotic strains against particular pathogens. For example, strains belonging to the genus *Ruegeria* showed little or no inhibition against *Vibrio* species but performed better against *T. maritimum*.

In this context, the possible specificity in antimicrobial properties suggests that combining strains could be a promising strategy. Synthetic Microbial Communities (SynComs), defined as artificial consortia of two or more microorganisms that perform functions of interest, have been extensively studied in humans [74], in terrestrial plants [75], and have recently been proposed in aquaculture, specifically in algae cultivation [76]. Applying this concept to octopus farming could offer important advantages. For instance, combining the high relative efficacy of *P. gallaeciensis*, the growth and inhibitory capacity of *Phaeobacter* sp. 4UAC3, and the specific activity of *Ruegeria* spp. could potentially generate a synergistic effect, thereby improving disease prevention and control in aquaculture systems. However, since some *Phaeobacter* strains may affect closely related members of the family Rhodobacteraceae [77], and it has been reported that *Phaeobacter* and *Ruegeria* colonize distinct ecological niches in aquaculture facilities [24], potential antagonistic interactions among candidate strains should be experimentally evaluated before designing synthetic consortia.

### 4.3. Effects of Phaeobacter sp. 4UAC3 Application During Early Octopus’ Development

With regard to the administration of probiotics in octopus farming during the early stages of development, *Phaeobacter* sp. 4UAC3 showed a strong capacity to colonize both the aquatic environment and the host. The strain persisted on octopus eggs for up to seven days without re-inoculation and remained detectable in paralarvae from 4 h after inoculation for up to six days when the assay finished, even after a change in environment. These results indicate that this bacterium is able to establish itself temporarily in both the culture system and the early life stages of *Octopus vulgaris*. However, the results also highlight the importance of the host’s developmental stage when applying probiotics. While the inoculation of *Phaeobacter* sp. 4UAC3 had a positive effect on paralarvae, in eggs, probiotic treatment significantly reduced the hatching rate compared with the control groups. During the final stages of embryonic development, eggs treated with *Phaeobacter* sp. 4UAC3 showed an accumulation of organic matter on their surface. This observation may have important implications due to the presence of organic deposits that could promote the growth of unwanted microorganisms and interfere with key physiological processes such as embryonic respiration, nutrient exchange and hatching [78,79]. It is important to note that these negative effects may not be caused by the probiotic itself, but also by methodological aspects of the experimental procedure (e.g., culture media addition). This suggests that the method of probiotic administration is also a critical factor. In particular, the centrifugation of the bacterial suspension may have carried over organic residues from the culture medium. When resuspended, these residues could have increased the organic load in the incubation bath and indirectly favored the growth of other microorganisms. This methodological limitation should therefore be considered when interpreting the results. For future studies, improvements in both culture and application protocols would be advisable. Performing extra centrifugation steps of the marine broth before probiotic cultivation could help minimize residual organic content and allow for a clearer evaluation of the effect of the bacterial strain on embryonic development. Alternatively, the use of inorganic media [30], although potentially limiting bacterial growth, could reduce the accumulation of organic matter in the system.

Considering the importance of the administration method, an alternative strategy to provide the probiotic to the paralarvae could involve the bioencapsulation of *Phaeobacter* in *Artemia*. In aquaculture systems, *Artemia* can act as a vector for pathogens [80]. Therefore, enriching them not only with microalgae to improve their nutritional value [81], but also with probiotic bacteria that antagonize *Vibrio* species present in their digestive tract, could represent an effective approach. This strategy could simultaneously reduce the risk of pathogen transmission through *Artemia* [82] and facilitate the delivery of *Phaeobacter* to octopus paralarvae through feeding. Short-term experiments have already demonstrated the feasibility of encapsulating Phaeobacter in Artemia [46,83]. However, further studies over longer periods are needed to evaluate the persistence of the probiotic after administration. In particular, it will be important to determine whether the probiotic is effectively transferred to the paralarvae after ingestion and whether it can survive passage through the gastrointestinal tract during digestion.

Finally, regarding larval survival, both the control group and the *V. lentus* group reached 100% mortality at 144 h, and their survival curves were very similar. While this result was expected for the *V. lentus* treatment, due to the previously documented pathogenicity of this bacterium [47,69], it was unexpected in the control group. This observation suggests that larvae may carry in most cases potential pathogenic or opportunistic bacteria that could be antagonized by *Phaeobacter*. Several characteristics of the culture system, including the use of a closed system, the small water volume, and limited oxygenation, could have negatively affected larval health and contributed to the high variability and mortality observed in both control and infected groups. In fact, the final objective of this study is not to achieve optimal survival of the paralarvae in culture (which would require experimental conditions incompatible with the proposed infection trials), but rather to assess, within a short time frame and within the limits of the experiment, the potential benefits of administering the probiotic. Therefore, despite the fact that mortality occurred at 6 days even in the probiotic group due to the reasons related to the aquarium conditions mentioned above, a positive effect of the probiotic is observed, reducing mortality. Larvae treated with *Phaeobacter* sp. 4UAC3 (both with and without *V. lentus*) survived until the end of the experiment in all replicates. Comparisons between *V. lentus* and *Phaeobacter*–*V. lentus*, as well as between the control and the *Phaeobacter* treatment, clearly showed significant differences in survival curves and markedly higher final survival when the probiotic was present. These results suggest that *Phaeobacter* may have mitigated the effects of suboptimal culture conditions and have limited the proliferation of opportunistic or pathogenic bacteria in the system, thereby contributing to improved survival of the paralarvae.

The variability observed between replicates is a well-recognized feature of in vivo infection challenge assays in marine larviculture and has been reported across a range of host–pathogen–probiotic combinations in aquaculture studies [48,84,85]. Such variability is generally attributed to small, uncontrolled differences in environmental conditions among replicate tanks that affect microbiome composition and host-microbe interactions.

The in vivo trials were designed to evaluate the probiotic effect of *Phaeobacter* sp. 4UAC3 from the egg to the paralarval stage. Paralarvae hatched from eggs previously treated with *Phaeobacter* were exposed again to the probiotic prior to pathogen challenge, as no probiotic was detected at this stage, and paralarvae hatched from untreated eggs were used as control. It can therefore be assumed that the protective effect observed in the survival analysis reflects the response to this direct paralarval inoculation, rather than a carryover effect from egg treatment. However, future experiments should evaluate the effect of *Phaeobacter* inoculation separately at each developmental stage.

Overall, these findings point to a potential protective effect of the probiotic. Future studies should investigate the mechanisms involved in the microbial dynamics, host–microbe interactions and potential ecological effects, for example, through metagenomic analysis. Histological analyses of paralarvae and transcriptomic studies, focusing on genes related to the immune response, would also contribute to elucidate underpinning mechanisms. Additionally, it is recommended to increase the number of replicates to enhance the statistical power; to perform long-term experiments, although this will be limited by the need to select an optimal diet that allows for proper growth and development during these early stages of life; and to consider the inclusion of clutches of different females to assess the progenitor variability effect. Furthermore, further experiments should also evaluate the probiotic under standard culture conditions, using optimized environmental and zootechnical parameters, rather than restricting the trials to small infection tanks of 0.5 L.

## 5. Conclusions

To sum up, these findings highlight the influence of parental care on the microbiome of octopus eggs and the potential of microbial manipulation by probiotic introduction as an alternative strategy in aquaculture. This study underlines the importance of carefully designing probiotic strategies that consider not only the bacterial strain or strains used, but also the developmental stage of the host and the method of probiotic application. As probiotic-treated eggs resulted in reducing hatching rates, *Phaeobacter* sp. 4UAC3 should be applied during the paralarval stage, promoting a significant increase in survival rates in infected paralarvae and having a positive effect on non-infected paralarvae despite variability. The positive effect in comparison with the control would suggest a beneficial effect in increasing paralarvae resilience. This work represents an initial step toward assessing how beneficial bacteria can be integrated into octopus aquaculture. By establishing an initial experimental framework, it opens the door to a more precise exploration and understanding of host–microbe interactions in octopus paralarvae rearing. Future research will be essential to advance this approach, particularly through studies that integrate multiple omic techniques to better unravel the dynamics between the host and its associated microbial communities.

## Figures and Tables

**Table 1:** Count and estimation of octopus’ eggs in each cluster.

	Counted Eggs	Estimated Eggs	Length of Principal Cluster (cm)	Maximum Length (cm)
Control 1	507	1014	7.055	7.988
Control 2 (*)	471	942	5.233	6.080
Treatment 1	477	954	6.176	6.978
Treatment 2 (*)	480	960	5.374	6.194
Treatment 3	180	360	2.324	2.933

(*) These clusters originated from the same initial cluster, which was divided into two.

**Table 2:** Estimation of the percentage of non-hatched eggs and the stage of embryonic development at which development stopped in non-hatched eggs. Average results are displayed with standard deviations.

	Non-Hatched Eggs (nº)	Non-Hatched Eggs (%)	Stage I–VIII (%)	Stage VIII–XVI (%)	Stage XV–XX (%)
Control 1	36	3.55	50	8.30	41.70
Control 2	32	3.40	65.60	12.50	21.90
Treatment 1	341	35.74	14.70	5.70	79.60
Treatment 2	177	37.23	34.00	4.30	61.70
Average Control		3.47 ± 0.11	57.81 ± 11.03	10.42 ± 2.97	31.77 ± 14.00
Average Treatment		36.49 ± 1.05	24.38 ± 13.65	4.97 ± 0.99	70.65 ± 12.66

## Data Availability

Data will be available upon request.

## Supplementary Materials

The following supporting information can be downloaded at https://www.mdpi.com/article/10.3390/microorganisms14051165/s1: Table S1. *p*-values obtained from the statistical analysis (Dunn’s test) performed on the inhibition halo areas produced by probiotics in the Well Diffusion Agar Assay. LRC4: *Ruegeria* sp. LRC4, ALR6: *Ruegeria* sp. ALR6, 4UAC3: *Phaeobacter* sp. 4UAC3, GAL: *Phaeobacter gallaeciensis*, 27/4: *Phaeobacter piscinae* 27/4. Table S2. *p*-values obtained from the statistical analysis (Dunn’s test) performed on the inhibition halo areas produced by probiotics in the Well Diffusion Agar Assay. LRC4: *Ruegeria* sp. LRC4, ALR6: *Ruegeria* sp. ALR6, 4UAC3: *Phaeobacter* sp. 4UAC3, GAL: *Phaeobacter gallaeciensis*, 27/4: *Phaeobacter piscinae* 27/4. Table S3. *p* values obtained from statistical analysis (Krskal Wallis and Dunn test) performed on the survival rates of paralarvae during the infection trial. C: Control, P: *Phaeobacter*, VL: *Vibrio lentus*, P_VL: *Phaeobacer*-*Vibrio lentus*. Table S4. *p* values from statistical analysis of survival curves using the Log-Rank test (Mantel–Cox). C: Control, P: *Phaeobacter*, VL: *Vibrio lentus*, P_VL: *Phaeobacer*-*Vibrio lentus*. Table S5. *p* values obtained from statistical analysis (Dunn Test) performed on total cultivable bacteria quantified in the paralarvae infection assay. C: Control, P: *Phaeobacter*, VL: *Vibrio lentus*, P_VL: *Phaeobacer*-*Vibrio lentus*. Figure S1. Well Diffusion Agar Assay of each probiotic *Ruegeria* sp. LRC4, *Ruegeria* sp. ALR6, *Phaeobacter* sp. 4UAC3, *Phaeobacter gallaeciensis*, *Phaeobacter piscinae* 27/4 against different pathogens: *Vibrio anguillarum*, *Vibrio lentus*, *Vibrio splendidus*, and *Tenacibaculum maritimum*. Figure S2. Inhibition halo/CFU ratio of each probiotic against different pathogens: *Vibrio anguillarum*, *Vibrio lentus*, *Vibrio splendidus*, and *Tenacibaculum maritimum*. LRC4: *Ruegeria* sp. LRC4, ALR6: *Ruegeria* sp. ALR6, 4UAC3: *Phaeobacter* sp. 4UAC3, GAL: *Phaeobacter gallaeciensis*, 27/4: *Phaeobacter piscinae* 27/4. Figure S3. Embryonic development of octopus eggs throughout the suspension bath trial with and without probiotics. Images taken with a calibrated stereoscopic magnifying glass.

## Abbreviations

The following abbreviations are used in this manuscript:

27_4	*Phaeobacter piscinae* 27/4
4UAC3	*Phaeobacter* sp. 4UAC3
ALR6	*Ruegeria* sp. ALR6
ASW	Artificial Seawater
CFU	Colony Forming Units
GAL	*Phaeobacter gallaeciensis*
IEO	Instituto Español de Oceanografía
IIM	Instituto de Investigaciones Marinas
LRC4	*Ruegeria* sp. LRC4
MA	Marine Agar
MB	Marine Broth
TDA	Tropodithietic acid
TSA	Tryptic Soy Agar
TSB	Tryptic Soy Broth
WDAA	Well Diffusion Agar Assay

## Appendix A

### Complementary Information About the Bacterial Strains Used

Probiotic strains:

- *Phaeobacter* sp. 4UAC3 (CECT 31030) was isolated from the thallus of *Ulva australis* collected in Tragove (Cambados), Ría de Arousa, Galicia, Spain (42°31′8″ N, 8°49′7″ W) in 2015.
- *Phaeobacter gallaeciensis* (CECT 7277) was obtained from the Spanish Type Culture Collection (CECT, Valencia, Spain).
- *Phaeobacter piscinae* 27-4 (CECT 7251) was obtained from the Spanish Type Culture Collection (CECT, Valencia, Spain). This strain was previously designated as *Roseobacter* sp. 27-4 [86]. Its inhibitory activity against pathogenic strains affecting turbot larvae has been described, particularly against *Vibrio anguillarum* [87]. In addition, it was used as a positive control due to its well-known antimicrobial activity [61].
- *Ruegeria* sp. ALR6 and *Ruegeria* sp. LRC4 were isolated at the Instituto de Investigaciones Marinas (IIM-CSIC) from cultures of turbot larvae based on their antagonistic activity against *Vibrio anguillarum* [88].

Pathogenic strains:

- *Vibrio anguillarum* (90-11-287, serotype O1) was originally isolated from rainbow trout [89] and provided by Prof. H. Birkbeck (Institute of Biomedical and Life Sciences, Glasgow, UK). This strain has been widely used in fish infection assays (salmonids and turbot) [87,88].
- *Vibrio lentus* was isolated from skin lesions of adult *Octopus vulgaris* specimens and provided by Dr. Farto (Department of Microbiology, University of Vigo, Spain). This strain has been used in cephalopod infection assays [90].
- *Vibrio splendidus* (LGP32) was provided by Prof. Philippe Roch (University of Montpellier, France) as a pathogen of the mussel *Mytilus galloprovincialis*. This strain has also been used in cephalopod infection assays [90].
- *Tenacibaculum maritimum* was isolated from diseased turbot in a fish farm in Galicia (Spain) and provided by Ana Riaza (Stolt Sea Farm, Merexo, Spain). This strain has been widely used in experimental assays [88].
