# Supplementary material for: Charting a Sustainable Course: Phaeobacter Inoculation as a Probiotic-Based Strategy for Common Octopus Aquaculture During Early Life Stages
Source: Microorganisms. 2026 May 21;14(5):1165. doi: 10.3390/microorganisms14051165 (PMC13209204; doi:10.3390/microorganisms14051165)
Supplement: Supplementary file 1 [file microorganisms-14-01165-s001.zip › microorganisms-4262147-supplementary.pdf]

SUPPLEMENTARY MATERIAL

Charting a Sustainable Course: *Phaeobacter* Inoculation as a Probiotic-Based Strategy for Common Octopus Aquaculture During Early Life Stages

Luana Granja [1,2], Gonzalo Del Olmo [1*], Jorge Carlos Santamaría [1], José Pintado [1] and Camino Gestal [2*]

[1] Integrated Marine Ecology (INMARE), Instituto de Investigacions Mariñas – CSIC, Rúa de Eduardo Cabello, 6, 36208 Vigo, Pontevedra, Spain. LG: luanagranjafz@gmail.com; GDO: gdelolmo@iim.csic.es; JCS: jsantamaria@iim.csic.es; JP: pintado@iim.csic.es

[2] Marine Molecular Pathobiology (PatoBioMar), Instituto de Investigacions Mariñas – CSIC, Rúa de Eduardo Cabello, 6, 36208 Vigo, Pontevedra, Spain. CG: cgestal@iim.csic.es

* Correspondence: gdelolmo@iim.csic.es; cgestal@iim.csic.es; Tel.: +34 986 23 19 30 ext 438674

Supplementary Tables

Supplementary Table S1. *P*-values obtained from the statistical analysis (Dunn's test) performed on the inhibition halo areas produced by probiotics in the Well Diffusion Agar Assay. LRC4: *Ruegeria* sp. LRC4, ALR6: *Ruegeria* sp. ALR6, 4UAC3: *Phaeobacter* sp. 4UAC3, GAL: *Phaeobacter gallaeciensis*, 27/4: *Phaeobacter piscinae* 27/4.

Bacterial isolated ID	Source	Color and morphology
Isolate 1	Without parental care	Brown
Isolate 2	Without parental care	Brown
Isolate 3	Without parental care	Brown
Isolate 4	Without parental care	Brown
Isolate 5	Without parental care	Brown
Isolate 6.1	Without parental care	Brown
Isolate 6.2	Without parental care	White – mucoid
Isolate 7	With parental care	Brown
Isolate 8	With parental care	Brown
Isolate 9	With parental care	Brown
Isolate 10	With parental care	Brown
Isolate 11	With parental care	Brown
Isolate 12	With parental care	Brown
Isolate 13	With parental care	Brown
Isolate 14	With parental care	Brown
Isolate 15	Without parental care	Yellow–orange – smaller colony
Isolate 16	Without parental care	White – dry irregular
Isolate 17	Without parental care	White – dry irregular
Isolate 18	Without parental care	Yellow–orange – irregular
Isolate 19	Without parental care	White – dry irregular
Isolate 20	Without parental care	Yellow–orange – larger colony
Isolate 21	With parental care	Brown – smaller colony

Supplementary Table S2. *P*-values obtained from the statistical analysis (Dunn's test) performed on the inhibition halo areas produced by probiotics in the Well Diffusion Agar Assay. LRC4: *Ruegeria* sp. LRC4, ALR6: *Ruegeria* sp. ALR6, 4UAC3: *Phaeobacter* sp. 4UAC3, GAL: *Phaeobacter gallaeciensis*, 27/4: *Phaeobacter piscinae* 27/4.

	Vibrio anguillarum	***Vibrio splendidus***	***Tenacibaculum maritimum***
27/4 – ALR6	-	-	$1.41 \cdot 10^{-4}$
27/4 – LRC4	1.000	-	0.308
4UAC3 – 27/4	0.023	-	0.866
4UAC3 – ALR6	-	-	0.024
4UAC3 – LRC4	0.024	0.024	1
GAL – 27/4	$2.64 \cdot 10^{-5}$	-	$3.46 \cdot 10^{-7}$
GAL – 4UAC3	0.036	0.036	$1.99 \cdot 10^{-4}$
GAL – ALR6	-	-	0.743

GAL – LRC4	$7.08{\cdot}10^{-7}$	$7.08{\cdot}10^{-7}$	0.008
LRC4 – ALR6	-	-	0.267

Supplementary Table S3. P values obtained from statistical analysis (Krskal Wallis and Dunn test) performed on the survival rates of paralarvae during the infection trial. C: Control, P: *Phaeobacter*, VL: *Vibrio lentus*, P_VL: *Phaeobacer-Vibrio lentus*.

		**24 h**	**48 h**	**72 h**	**144h**
Kruskal-Wallis		0.392	0.434	0.036	0.022
Dunn Test	C - P	0.472	0.474	0.178	0.138
	C - P_VL	1.000	1.000	0.137	0.055
	P - P_VL	0.472	1.000	1.000	1.000
	C - VL	1.000	1.000	1.000	1.000
	P - VL	0.472	0.474	0.104	0.138
	P_VL - VL	1.000	1.000	0.078	0.055

Supplementary Table S4. P values from statistical analysis of survival curves using the Log-Rank test (Mantel–Cox). C: Control, P: *Phaeobacter*, VL: *Vibrio lentus*, P_VL: *Phaeobacer-Vibrio lentus*.

	C	P	P_VL
P	$1.60{\cdot}10^{-9}$		
P_VL	$2.25{\cdot}10^{-9}$	0.911	
VL	0.715	$2.17{\cdot}10^{-10}$	$2.17{\cdot}10^{-10}$

Supplementary Table S5. P values obtained from statistical analysis (Dunn Test) performed on total cultivable bacteria quantified in the paralarvae infection assay. C: Control, P: *Phaeobacter*, VL: *Vibrio lentus*, P_VL: *Phaeobacer-Vibrio lentus*.

		**-4 h**	**0 h**	**24 h**	**48 h**	**144h**
Total Culturable Bacteria	C - P	1.000	0.118	0.071	0.467	-
	C - P_VL	1.000	0.118	0.028	1.000	-
	P - P_VL	1.000	1.000	1.000	0.212	0.138
	C - VL	1.000	1.000	0.639	1.000	-
	P - VL	1.000	0.118	0.925	1.000	-
	P_VL - VL	1.000	0.118	0.523	1.000	-

Supplementary Figures

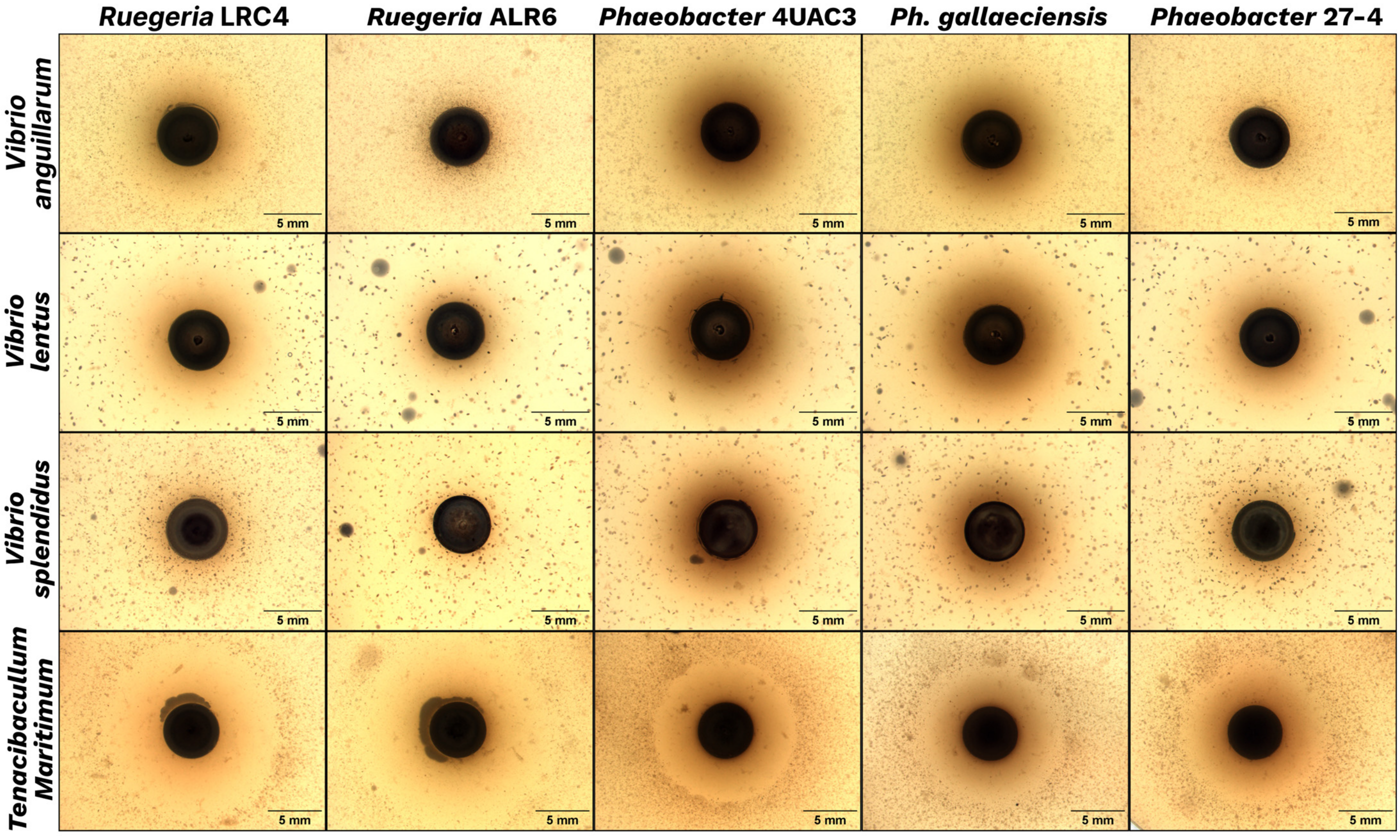

Supplementary Figure S1. Well Diffusion Agar Assay of each probiotic *Ruegeria* sp. LRC4, *Ruegeria* sp. ALR6, *Phaeobacter* sp. 4UAC3, *Phaeobacter gallaeciensis*, *Phaeobacter piscinae* 27/4 against different pathogens: *Vibrio anguillarum*, *Vibrio lentus*, *Vibrio splendidus*, and *Tenacibaculum maritimum*.

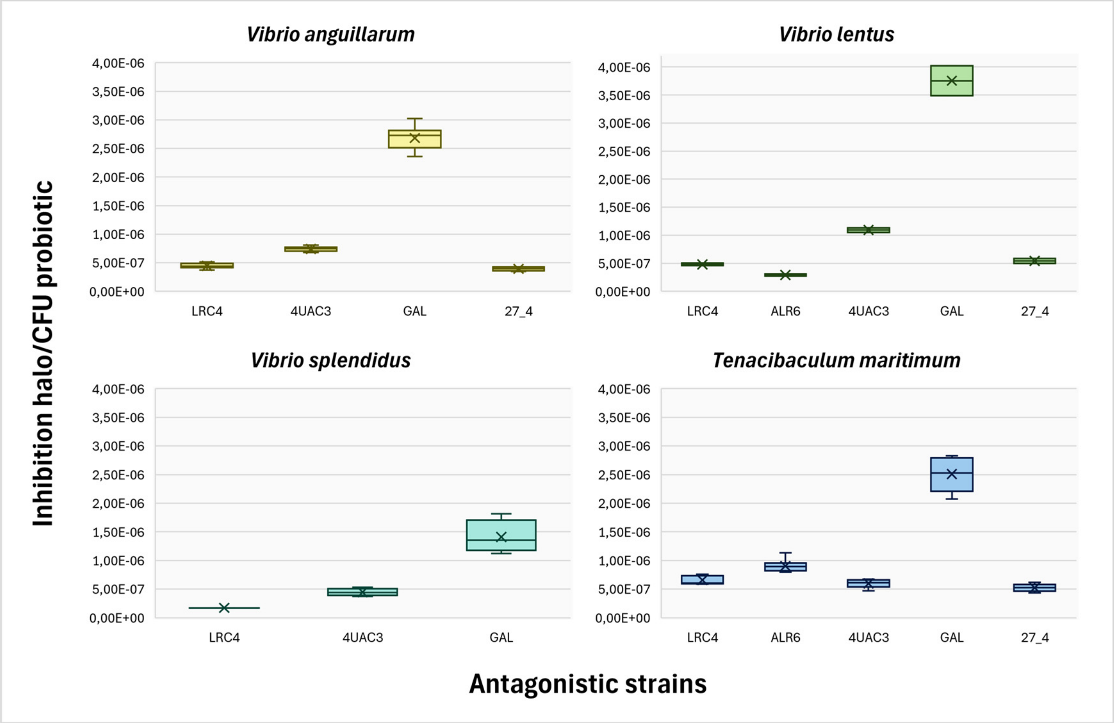

Supplementary Figure S2. Inhibition halo/CFU ratio of each probiotic against different pathogens: *Vibrio anguillarum*, *Vibrio lentus*, *Vibrio splendidus*, and *Tenacibaculum maritimum*. LRC4: *Ruegeria* sp. LRC4, ALR6: *Ruegeria* sp. ALR6, 4UAC3: *Phaeobacter* sp. 4UAC3, GAL: *Phaeobacter gallaeciensis*, 27/4: *Phaeobacter piscinae* 27/4

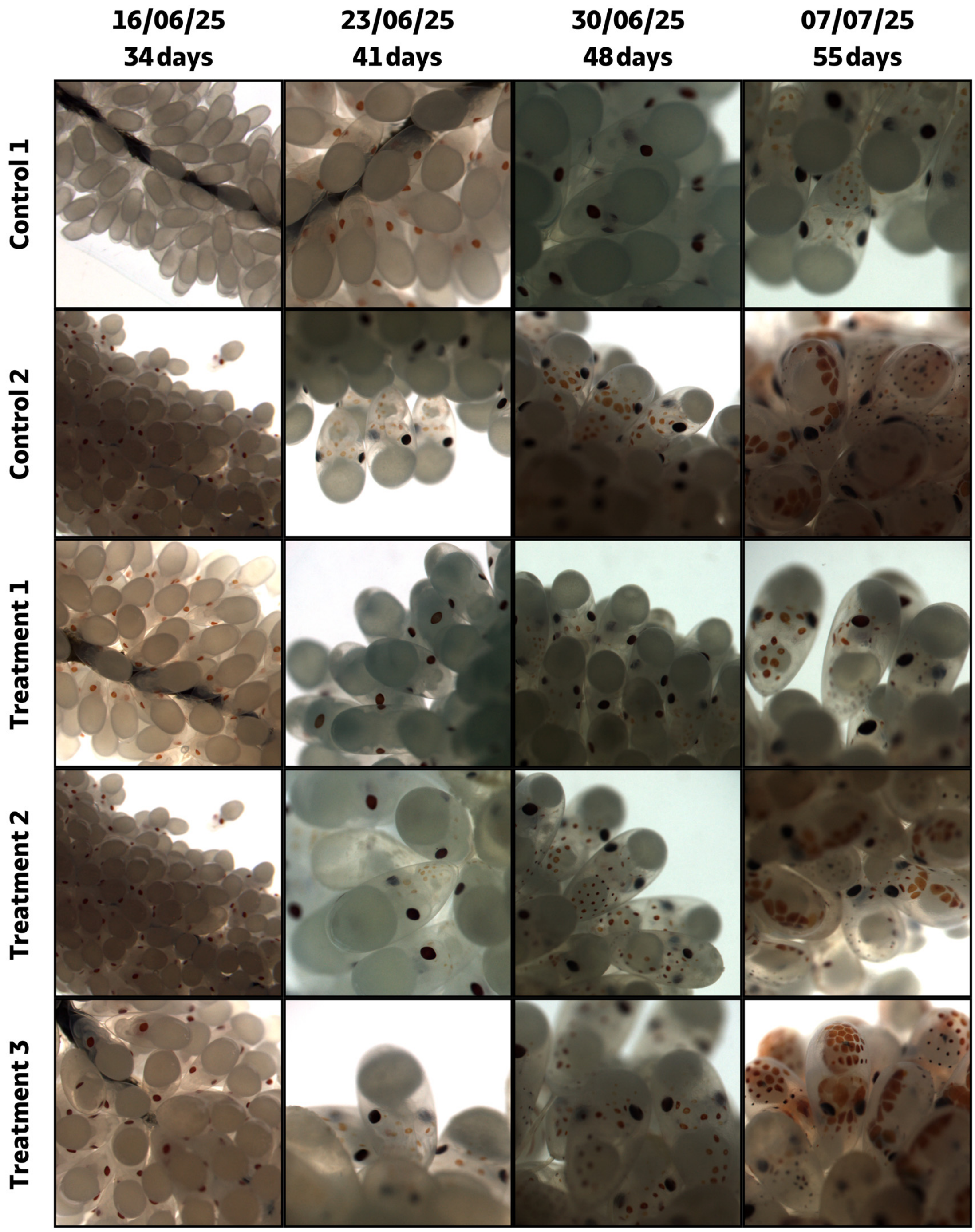

Supplementary Figure S3. Embryonic development of octopus eggs throughout the suspension bath trial with and without probiotics. Images taken with a calibrated stereoscopic magnifying glass.
